# Symmetry-breaking-induced off-resonance second-harmonic generation enhancement in asymmetric plasmonic nanoparticle dimers

**DOI:** 10.1515/nanoph-2024-0118

**Published:** 2024-06-03

**Authors:** Yaorong Wang, Zhiwei Peng, Yannick De Wilde, Dangyuan Lei

**Affiliations:** Department of Materials Science and Engineering, 53025 City University of Hong Kong, Hong Kong S.A.R., China; Institut Langevin, ESPCI Paris, CNRS, Université PSL, Paris, France; Hong Kong Branch of National Precious Metals Material Engineering Research Centre, 53025 City University of Hong Kong, Hong Kong S.A.R., China

**Keywords:** second-harmonic generation, nanoparticle dimer, symmetry breaking, plasmon hybridization, nonlinear optics

## Abstract

The linear and nonlinear optical properties of metallic nanoparticles have attracted considerable experimental and theoretical research interest. To date, most researchers have focused primarily on exploiting their plasmon excitation enhanced near-field and far-field responses and related applications in sensing, imaging, energy harvesting, conversion, and storage. Among numerous plasmonic structures, nanoparticle dimers, being a structurally simple and easy-to-prepare system, hold significant importance in the field of nanoplasmonics. In highly symmetric plasmonic nanostructures, although the odd-order optical nonlinearity of the near-surface region will be improved because of the enhanced near-fields, even-order nonlinear processes such as second-harmonic generation (SHG) will still be quenched and thus optically forbidden. Under this premise, it is imperative to introduce structural symmetry breaking to realize plasmon-enhanced even-order optical nonlinearity. Here, we fabricate a series of nanoparticle dimers each composed of two gold nanospheres with different diameters and subsequently investigate their structural asymmetry dependent linear and nonlinear optical properties. We find that the SHG intensities of gold nanosphere dimers are significantly enhanced by structural asymmetry under off-resonance excitation while the plasmonic near-field enhancement mainly affects SHG under on-resonance excitation. Our results reveal that symmetry breaking will play an indispensable role when designing novel coupled plasmonic nanostructures with enhanced nonlinear optical properties.

## 1 Introduction

In the past two decades, plasmonic metal nanoparticles have attracted widespread attention due to their potential applications in catalysis, clinical diagnostics, optical data storage, etc. [1], [2], [3], [4], [5], [6] Among different nanostructures, nanoparticle dimers composed of two nanospheres are promising candidates for generating huge electromagnetic fields in their gap region [7], [8], [9]. The nanosphere dimers possess rich plasmonic properties and are easy to fabricate, which makes them an appropriate platform for studying linear and nonlinear optical phenomena. Furthermore, the plasmonic modes of the dimer originate from the near-field interaction between the strongly localized surface plasmon resonance (LSPR) of each nanosphere, which can be modulated easily and efficiently by modifying the nanosphere size or gap distance [10].

The tightly confined electromagnetic field in the gap of nanosphere dimers promotes nonlinear optical effects such as second-harmonic generation (SHG), which makes them an excellent platform to study and control nonlinear light–matter interactions at the nanoscale [11], [12], [13], [14], [15], [16]. The radiation at the double frequency 2*ω* is produced by the nonlinear polarization *P*(2*ω*) generated by the interaction between the fundamental electric field *E*(*ω*) and the medium. Two photons at the fundamental frequency *ω* are converted into one photon at the second harmonic (SH) frequency 2*ω*, which can be described as [17], [18]

(1) $$P\left(2\omega \right)=\chi^{\left(2\right)}:E\left(\omega \right)E\left(\omega \right)$$

where $\chi^{\left(2\right)}$ is the second-order nonlinear susceptibility tensor. Similarly, applying the space inversion operation to the system, we have

(2) $$\begin{array}{ll} -P\left(2\omega \right) & =\chi^{\left(2\right)}:\left(-E\left(\omega \right)\right)\left(-E\left(\omega \right)\right)\\ & =\chi^{\left(2\right)}:E\left(\omega \right)E\left(\omega \right) \end{array}$$

From the Equations (1) and (2), it can be seen that in the dipole approximation, the SHG emission is forbidden in a centrosymmetric medium, $P\left(2\omega \right)=-P\left(2\omega \right)=0$ .

So far, the research on plasmonic dimers mainly focuses on metallic nanoparticles made of gold, silver, and copper, where SHG is forbidden in the bulk because of the microscopically centrosymmetric arrangement of their atoms. As such, observed SHG signals are ascribed to (1) surface dipolar susceptibility, and (2) bulk quadrupole contribution originating from the retardation effect [19], [20]. Generally, the SHG output of metallic nanoparticles is highly sensitive to both their shape and size. The nonlinear-optical conversion efficiency can be significantly impacted by shape imperfections that introduce local symmetry breaking [21], [22], potentially leading to enhanced SHG [23]. Furthermore, nanoparticles made of non-centrosymmetric materials demonstrate efficient SHG [24] which can be used for nonlinear optical microscopy [25], nonlinear phase control [26] and sensing [27].

The concept of SHG sensitivity to the local symmetry can be naturally extended to the structural mesoscopic symmetry of plasmonic nanoobjects. Indeed, in symmetric gold nanoparticle dimers, though LSPR strongly enhances local electric fields and thus nonlinear polarization in the near field [16], [28], the far-field SHG emission is limited by the structural symmetry of the dimers. However, once the symmetry is broken, the cancellation is incomplete resulting in the generation of SH light [29], [30], [31], [32], [33]. In our previous investigation, we delved into a nanoparticle-on-film configuration as an extreme asymmetric case, contrasting with a symmetric dimer [33]. We observed that, under the same excitation and collection conditions, the SHG intensity of a gold nanosphere-on-film system exceeds that of a weakly asymmetric gold nanosphere dimer by more than 13-fold. In this work, our principal focus lies in the systematic exploration of SHG within aymmetric dimers, where the emphasis is placed on the gradual modulation of structural symmetry. To reflect the mesoscopic symmetry of the nanostructure, we introduce an effective nonlinear susceptibility denoted as $P\left(2\omega \right)=\chi_{\text{eff}}^{\left(2\right)}:E\left(\omega \right)E\left(\omega \right)$ . Here, *E*(*ω*) refers to the LSPR-enhanced electric field in the plasmonic hotspot, and $\chi_{\text{eff}}^{\left(2\right)}=0$ in symmetric dimers. We analyze the SHG originating in the geometric symmetry breaking in Au nanosphere dimers in both off- and on-resonant conditions. Performing nonlinear micro-spectroscopy of single dimers allows us to study the impact of gradually increased structural asymmetry on the far-field SHG intensity. We focus on individual nanosphere dimers rather than regular arrays, thereby eliminating any potential influence arising from collective effects, such as plasmonic surface lattice resonance (SLR) and other analogous phenomena [34]. We outline symmetry breaking and plasmon-driven near-field enhancement as two principal mechanisms responsible for the observed SHG increase with the dimer asymmetry. Although the near-field enhancement still dominates the SHG emission under the on-resonant excitation condition, the SHG intensities of gold nanoparticle dimers can be significantly enhanced by structural asymmetry under the off-resonant condition.

## 2 Results and discussion

**Figure 1:**
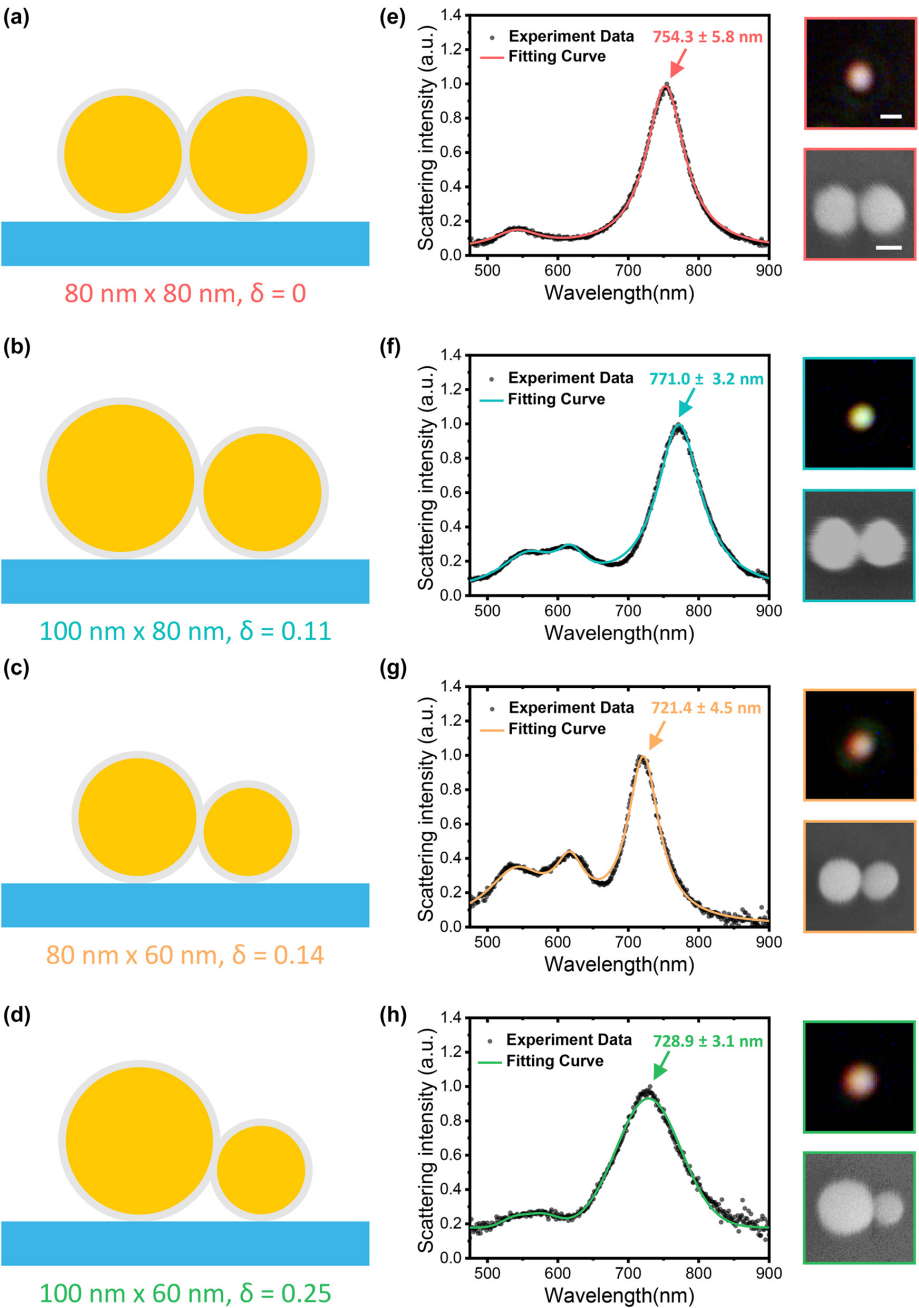
Linear optical properties of dimers with varied degree of structural asymmetry. (a–d) Schematic illustrations of fabricated symmetric and asymmetric dimers, where the diameters of the two spheres and their degree of asymmetry *δ* are labeled. (e–h) Measured DF scattering spectra of dimers with different degrees of asymmetry, where the peak position of each LBDP mode and its error bar are marked. The solid lines are Lorentz fits to the scattering spectra (black dots). The scale bar in the DF image is 500 nm, and in the SEM image is 50 nm.

The dimers used in this paper are prepared on Si/SiO_2_ substrates. In a dimer, the two spherical nanoparticles are separated by a nanosize gap hosting alkanedithiol molecules [10], [35], [36], [37]. The advantage of Au–S bonds for realization of the dimer assembly is that the gap distance can be steadily controlled and remains homogeneous (see Section S1). In this work we find the gap width of about 1.3 nm, resulting in a strong localization and enhancement of the electromagnetic fields. Figure 1(a)–(d) shows the geometry of the prepared gold nanodimer. The degree of asymmetry of a dimer is defined by *δ* = |*d*_1_ − *d*_2_|/(*d*_1_ + *d*_2_) where *d*1,2 are the diameters of the two nanospheres. All dimers are assembled from 60 nm, 80 nm, and 100 nm gold spheres (see Figure 1(a)–(d)) with *δ* = 0, 0.11, 0.14, and 0.25 (color-coded red, blue, orange, and green, respectively). Figure 1(e)–(h) show the measured dark-field (DF) scattering spectra of individual dimers with different symmetries under unpolarized excitation. A single stronger peak originating from the longitudinal bonding dipolar plasmon (LBDP) mode occurs between 700 nm and 800 nm, varying with the structural parameters of dimers [38]. The dimer’s DF images and scanning electron microscopy (SEM) images are also shown alongside the DF spectra to better illustrate the scattering pattern and morphology of the nanosphere dimer with different symmetries. We specify the resonant wavelength of LBDP mode on the scattering spectra obtained from experiments with the error bar obtained from multiple measurements (see Figure S1 in Supporting Information). Single-particle optical characterization allowed us to exclude monomers and randomly formed oligomers from the target dimers (Figure S3(b) in Supporting Information) [39].

To better understand the nature of the modes of scattering peaks of the dimer, we further analyzed the plasmon hybridization modes by adding a polarizer in the collection optical path (Figure S3(a) in Supporting Information). It is generally accepted that plasmon coupling can be considered analogous to molecular hybridization [40], [41]. The dimer plasmons can be regarded as a combination of bonding and anti-bonding, that is, as a hybridization of single nanoparticle plasmons. The plasma modes of the two particles in the dimer (Ψ_1_ and Ψ_2_) can hybridize either in-phase (Ψ_1_ + Ψ_2_) or out-of-phase (Ψ_1_ − Ψ_2_). When the optical field is polarized along the inter-particle axis, i.e. longitudinal polarization, the in-phase hybridization mode reflects the bonding mode (denoted as *σ*), the electric field in the gap of the dimer is enhanced, and the LSPR frequency is red-shifted. The out-of-phase hybridization mode is the anti-bonding mode (denoted as *σ* ^∗^), with the electric field along the short axis of the dimer and a blue-shifted LSPR frequency [41]. Conversely, when the polarization is along the short axis of the dimer, i.e. transverse polarization, the in-phase hybridization mode is an anti-bonding mode (*π* ^∗^), while the out-of-phase hybridization mode represents the bonding mode (*π*).

**Figure 2:**
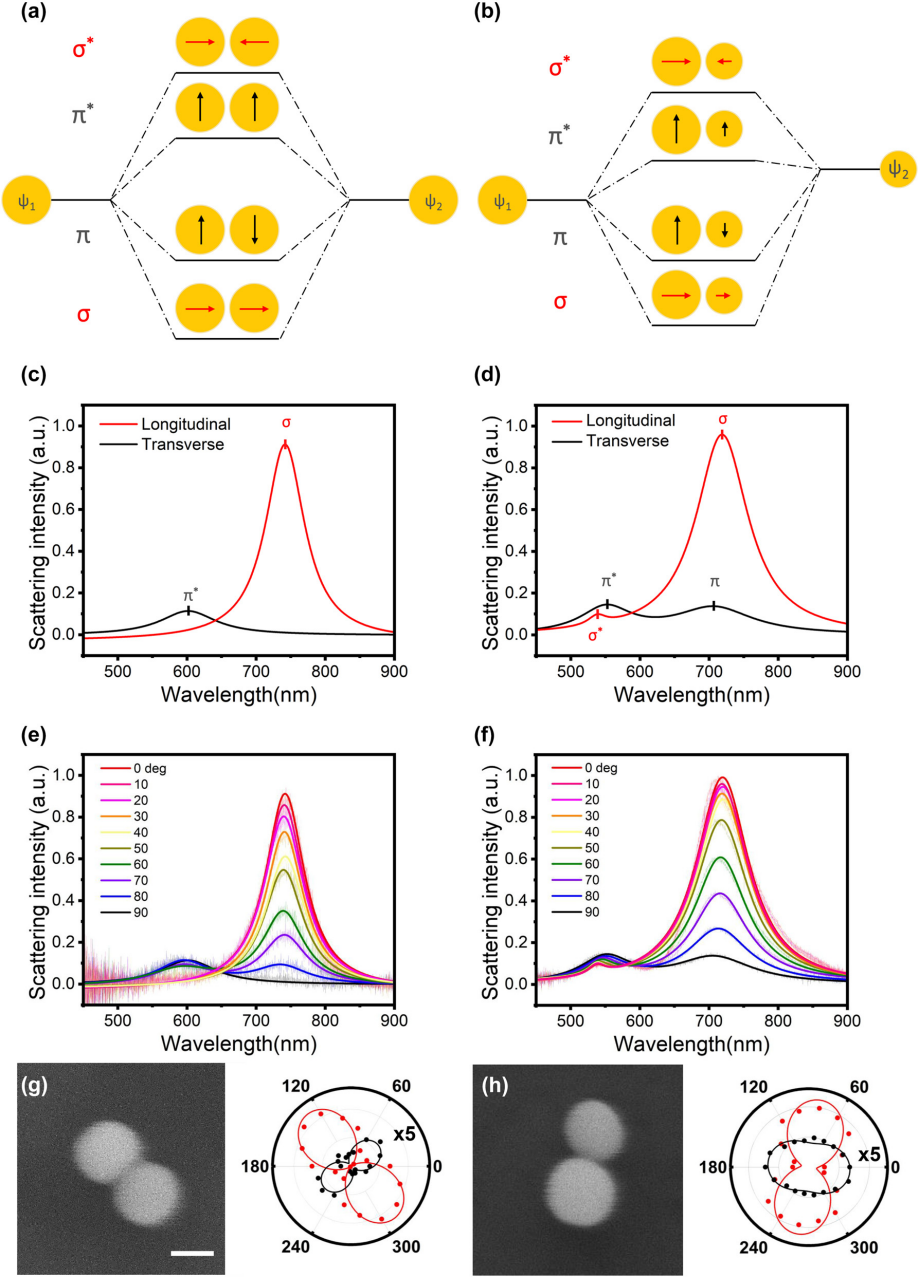
Linear optical properties of symmetric and asymmetric gold nanoparticle dimer. (a, b) Plasmon hybridization models for a symmetric dimer (a) and an asymmetric dimer (b). (c, d) Transverse (black line) and longitudinal (red line) coupled modes in a symmetric gold nanoparticle dimer (c), where the in-phase mode is the only bright mode that can be optically detected in the far field. In contrast, an active out-of-phase mode is observed in the asymmetric dimer (d). These spectra are derived from Lorentz fitting of experimental data. (e, f) Scattering spectra of a symmetric (e) and asymmetric (f) gold nanoparticle dimer at intermediate polarization angles between longitudinal mode (0°) and transverse mode (90°). The more transparent lines in the background are experiment data, and the solid curves are obtained by Lorentz fitting. (g, h) Measured (dots) and fitted (line) polar plots of polarization-resolved scattering intensities for the transverse (black) and longitudinal (red) modes in the symmetric (g) and asymmetric (h) dimer, with their intensity maximum axes in good agreement with the dimer orientation shown in SEM micrographs. The scale bar in the SEM image is 50 nm. The transverse mode (black) is enlarged by 5 times for better display.

Figure 2(a) and (b) demonstrate the typical plasmon hybridization models of symmetric (*δ* = 0) and asymmetric (*δ* = 0.14) gold nanosphere dimers in this work. Consistent with the predictions of the plasmonic hybridization model, the scattering spectra of the symmetric dimers show two distinct modes. By rotating the polarizer in the collection optical path, the scattering spectra of the longitudinal and transverse polarization can be obtained. The Lorentz fitting curves of experimental results for these two cases are plotted in Figure 2(c). It can be seen that, whether it is longitudinal or transverse coupling, only the in-phase mode is optically allowed. In all intermediate angular orientations, the scattering spectrum is a linear combination of the two orthogonal modes [42], shown in Figure 2(e) as a function of the polarizer angle. Although it is not possible in optical scattering experiments to independently know the angle of the polarizer relative to the inter-particle axis, we were able to assign the two modes through theoretical analysis [41]. Please note that in order to demonstrate the difference between symmetrical and asymmetrical dimers, here we uniformly set the inter-particle axis to 0° instead of the real reading of the polarizer.

Likewise, Figure 2(d) shows the scattering spectra of longitudinal and transverse polarization of the asymmetric dimer. Both spectra contain two modes. This observation contrasts with the symmetric dimer, as expected according to the plasma hybridization model shown in Figure 2(b). The in-phase hybridization (Ψ_1_ + Ψ_2_) and the out-of-phase hybridization (Ψ_1_ − Ψ_2_) can be observed simultaneously, regardless of the longitudinal and transverse polarizations. That is, *σ* ^∗^ and *π*, which are dark in symmetric dimers, turn bright in asymmetric dimers, because of an incomplete cancellation of the dipoles of the two different size particles [43]. In the weak coupling regime, the intensity of the out-of-phase mode will be much weaker than that of the in-phase mode. Similarly, Figure 2(f) shows the scattering spectra of an asymmetric dimer at intermediate angles between the longitudinal and transverse polarizations.

The scattering intensity at the LSPR wavelength changes when rotating the polarizer, allowing us to determine the exact orientation of the dimer and the relative efficiency of the excitation of the LSPR modes. In Figure 2(g) and (h), the scattering intensities of the bonding mode (red) and anti-bonding mode (black) of the symmetric and asymmetric dimers are shown as a function of polarization angle in polar coordinates. In a good agreement with the SEM characterization, the scattering efficiency exhibits a maximum at an axis angle of about ∼130° for the symmetric dimer and about ∼80° for the asymmetric dimer due to their respective longitudinal bonding dipolar plasmon (LBDP) mode along the dimer axis [44], [45], [46]. The data associated with the transverse anti-bonding dipolar plasmon (TADP) mode, where the excited dipole moment is directed perpendicular to the dimer axis, is magnified five times for ease of display. The typical dipole-like pattern observed in experiments can be fitted by the cosine-square function, as shown by the dots and lines in Figure 2(g) and (h). The degree of polarization (DoP) defined as

(3) $$\text{DoP}=\frac{I_{\max}-I_{\min}}{I_{\max}+I_{\min}}$$

can be used to quantitatively describe the dipole-like pattern polarization, where *I*_max_ (*I*_min_) is the maximum (minimum) intensity parallel (perpendicular) to the dipole axis. In our case, a stronger linear polarization response was observed for the symmetric dimer (*δ* = 0, DoP of 0.8 and 0.97 for TADP and LBDP, respectively) than for the asymmetrical dimer (*δ* = 0.14, DoP of 0.27 for TADP and 0.76 for LBDP). Furthermore, as the *δ* continued to increase, a decrease in the DoP was observed (*δ* = 0.25, the DoP of TADP is 0.39, and the DoP of LBDP is 0.68). The noticeable reduction of DoP in asymmetric dimers indicates the non-negligible coupling between the plasmon modes. Originating in the geometric symmetry breaking, this coupling can be a fingerprint of the mode hybridization, modulating the distribution of the electric field in the gap. In turn, the latter is key for the asymmetry driven SHG response of the nanosphere dimers, as we discuss below.

For qualitatively understand the modulation mechanism of symmetry breaking on the SHG, we have numerically analyzed nanosphere dimers with different symmetries. The permittivity of gold nanosphere was taken from the empirical data given by Johnson and Christy [47]. The dielectric permittivity of alkanedithiol, similar to that of polyethylene, is approximately 2.25 [48], [49], [50], [51], [52]. The simulations are based on a phenomenological free electron model (see Section S2). The fundamental light polarized along the main dimer axis excites the LBDP mode. For centrosymmetric metals, the main component of the nonlinear susceptibility tensor $\chi_{⊥⊥⊥}^{\left(2\right)}$ originates from the metal surface [53].

**Figure 3:**
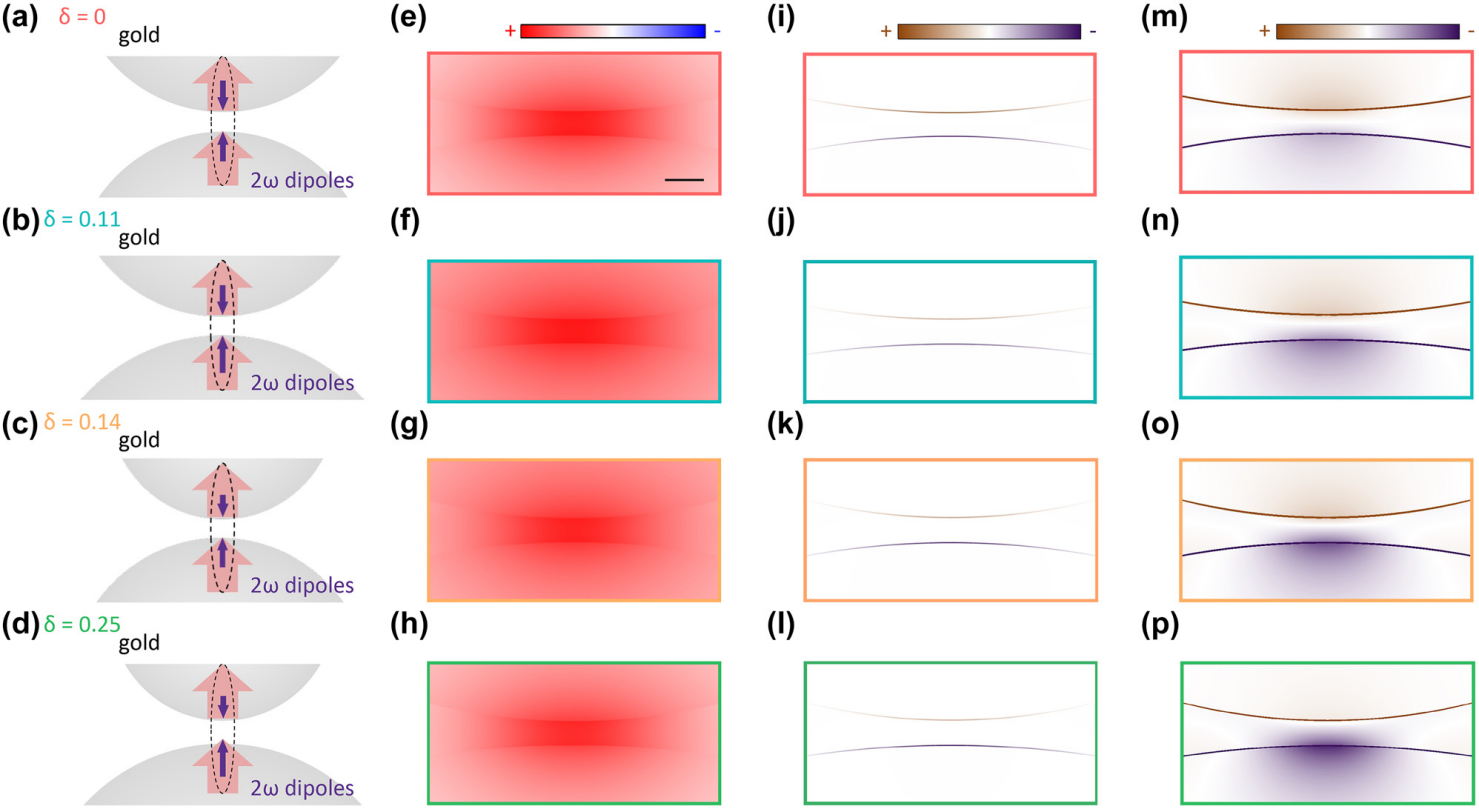
Analysis of the SH origin in gold nanosphere dimers with different symmetry conditions. (a–d) Schematics of the SH origin in the nanosphere dimers with varying degrees of asymmetry *δ*. Red thick arrows indicate the dipolar response to the driving field oriented along the nanogap axis, *E*⊥. Purple arrows represent the induced 2*ω* dipoles responsible for the far-field SH radiation. (e–h) Simulated near-field polarization distributions, *P*_*z*,2*ω*_, at the fundamental wavelength of 900 nm for the structures in (a–d) with a gap *g* = 1.3 nm. The Scale bar in (e) is 2 nm. (i–l) Calculated near-field polarization distributions, $P_{z,2\omega }^{0}$ , at the SH wavelength induced by the respective fundamental fields (Eqation (1)), which are significant only at the interface of the centrosymmetric metal. (m–p) SH polarization distributions resulting from the non-resonant local surface plasmon (LSP) coupling of the surface nonlinear sources in (i–l) to the dimers.

For the symmetric dimer shown in Figure 3(a), the SH dipole moments are equal at both sides of the nanogap but point in the opposite directions. As such, the far-field SHG radiation is quenched by the destructive interference of the opposite dipoles, as shown in Figure 3(i) and (m). In the case of several asymmetric dimers shown in Figure 3(b)–(d), the SH dipole moments still demonstrate an anti-bonding distribution, but the structural asymmetry results in their imbalance leading to the non-vanishing far-field SHG output. The fundamental frequency field (Figure 3(e)–(h)) is driven by an incident plane wave, resulting in the field enhancement inside the gap. The SH field (Figure 3(m)–(p)) is driven by nonlinear polarization source at the metal surface [54]. Symmetry breaking in an asymmetric dimer occurs through two aspects: (1) local SHG polarization ( $P_{z,2\omega }^{0}$ in Figure 3(j)–(l)) has an asymmetric distribution on the surface; (2) global SHG polarization ( $P_{z,2\omega }^{\text{LSP}}$ in Figure 3(n)–(p)). Because of the larger surface area, the nonlinear polarization is always stronger on the larger sphere than that on the smaller one.

**Figure 4:**
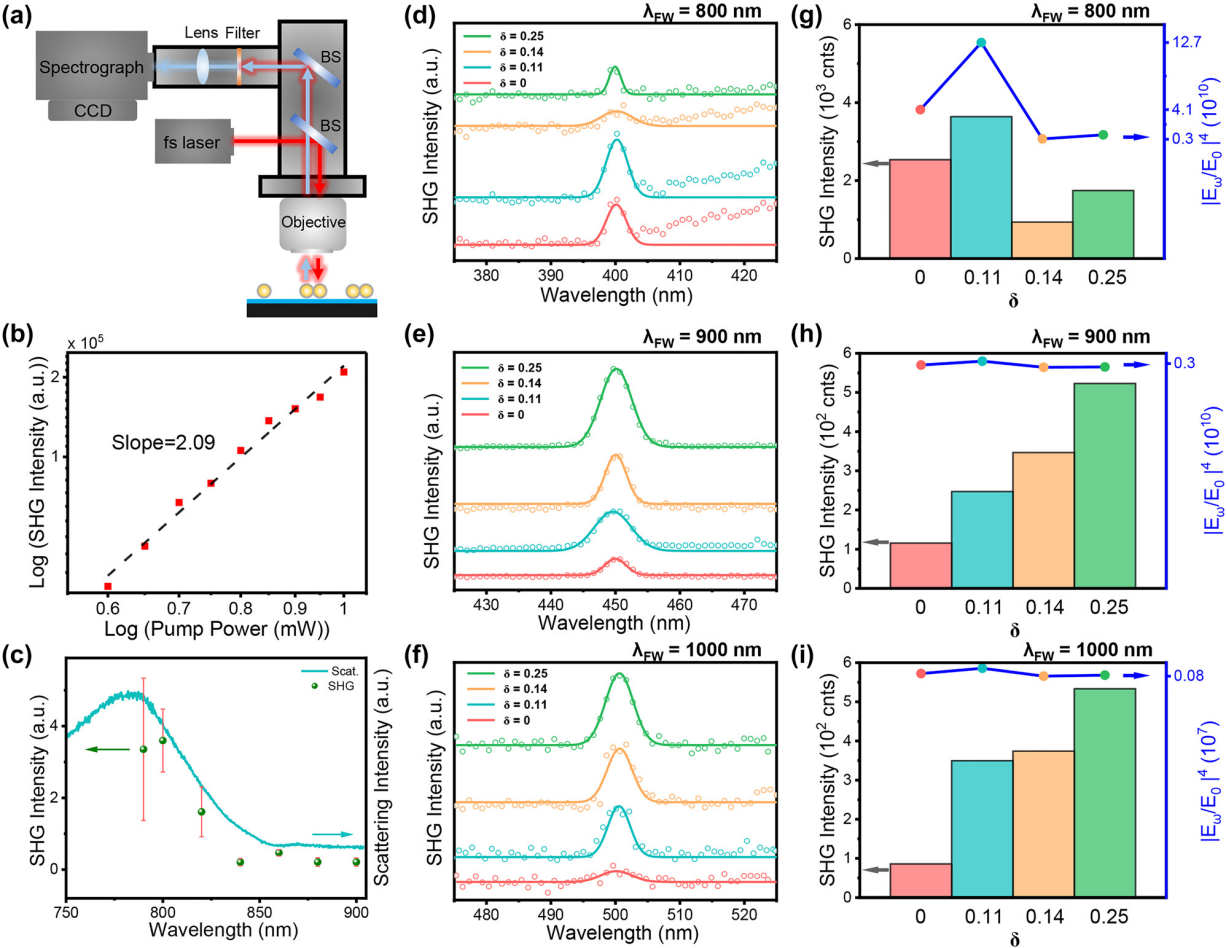
Nonlinear-optical properties of dimers with varied degrees of asymmetry. (a) Schematic diagram of experimental setup. (b) SHG peak intensities as a function of pump power in a log-log plot. (c) Excitation wavelength dependent SHG intensities measured for the dimer with δ = 0.11 (green dots). The linear scattering spectrum is shown for comparison. SH emission spectra of four gold nanosphere dimers with varied degree of structural asymmetry, measured at excitation wavelengths of 800 nm (d), 900 nm (e), and 1000 nm (f), respectively. The circles are experimental data points, and the solid lines represent Gaussian fitting. Under the on-resonance excitation at 800 nm, the SHG peak intensity obtained by fitting (histogram, left axis) shows consistency with the calculated near-field enhancement at the excitation wavelength (dots and line, right axis), as shown in (g). Under off-resonance excitation wavelengths of 900 nm (h) and 1000 nm (i), the higher the degree of structural asymmetry, the larger the SHG peak intensity.

In our experiments, the output of a femtosecond laser (Chameleon, 140 fs pulse duration, 80 MHz repetition rate) at fundamental wavelengths of 800 nm, 900 nm, and 1000 nm was focused on the sample by a high numerical aperture microscope objective (MPlanApo 480 100×, Olympus, NA = 0.95), as shown in Figure 4(a). The SHG emitted by the dimers is collected with the same objective and then reflected into the spectrograph by two beam splitters (BS). A 600 nm short-pass filter was inserted in the detection path, which removed the pump laser and allowed the SHG signal to pass. We were unable to observe SHG from a gold monomer under the average input power (∼600 µW) used in the experiments due to the lack of symmetry breaking, as described above.

We measured SHG response of dimers with different degree of asymmetry *δ* while maintaining excitation at 800 nm wavelength, as shown in Figure 4(d). Employing the fundamental radiation polarized along the main axis of the dimers, we were able to resonantly excite the LBDP mode providing the strongest enhancement of the electric field in the dimer gap. For different *δ*, a prominent SHG peak in the spectrum is observed at 400 nm wavelength, accompanied by broad two-photon luminescence (TPL) with comparable intensity. The quadrupole contribution is responsible for the non-zero SHG output of the dimer with *δ* = 0 in our experiments, indicating stronger field gradients in the dimer system [55], [56]. Additionally, slight asymmetries due to size dispersion of the nanospheres may also contribute to the observed weak SHG. Figure 4(b) present the log-log plots of SHG signals as a function of the pump power. The linear fits to the power-dependent evolutions reveal slopes of ∼2, confirming the second-order nature of SHG processes. Figure 4(g) shows SHG intensity at different *δ* as histograms. Contrary to our previous expectations, the largest SHG was not observed when *δ* was the largest.

To understand this behavior, we calculated the near-field enhancement of the electric field in the gap of dimers with different *δ*. When the particle size is changed, the LBDP resonance of dimers shifts, and respective near-field enhancement at 800 nm changes. It can be seen from Equation (1) that the SHG intensity is proportional to the fourth power of the locally enhanced field inside the dimer gap. We note that the plasmon-induced field enhancement at the double frequency can be ignored due to the strong contribution of the interband transitions in Au below the ∼530 nm wavelength. In Figure 4(g), the histogram shows the SHG intensities of dimers with different *δ*, while the corresponding calculated fourth power of the electric field enhancement inside the gap at the wavelength of 800 nm is shown with dots. This result is qualitatively consistent with the observation in the experiment: the dimer with *δ* = 0.11 which exhibits the strongest SHG has the strongest near-field enhancement (|*E*_*ω*_/*E*_0_|^4^) at 800 nm, two orders of magnitude more than the dimer with *δ* = 0.14. This also matches the wavelength shift in LBDP resonance. It indicates that the SHG of the dimer originates from the dimer gap and is modulated by the near-field enhancement there under on-resonant excitation.

To clarify the influence of electric field enhancement in SHG of nanosphere dimer, we also examined the dependence of the SHG intensity on the fundamental wavelength (FW) for a dimer with *δ* = 0.11, as shown in Figure 4(c). It can be seen that the strongest SHG was indeed generated at the LBDP resonance (∼790 nm) of the dimer with *δ* = 0.11 where the maximum electric field enhancement is achieved, in good agreement with the prediction based on the numerical simulation (Figure S3(b) in Supporting Information). As the wavelength increases, the *λ*_FW_ moves away from the LDBP resonance, and a rapid decrease in SHG intensity is observed. This indicates that near-field enhancement plays a crucial role in the SHG of gold nanosphere dimer.

However, in the off-resonant case, geometric asymmetry primarily contributes to the SHG of the dimers. In Figure 4(e), the SHG spectra of the dimers excited at *λ*_FW_ = 900 nm are presented. It can be seen that as the degree of asymmetry *δ* increases, the SHG intensity of the dimer also increases. This observation holds true at *λ*_FW_ = 1000 nm too in Figure 4(f), which is even farther away from the LBDP resonance. It is worth noting that under off-resonant conditions, the near-field enhancement for both fundamental wavelength is consistently low and in a comparable range (seen in Figure 4(h) and (i)), which is several orders of magnitude smaller than that under on-resonant case. Therefore, in the case of off-resonant excitation, the SHG enhancement is dominated by symmetry breaking rather than near-field enhancement. Overall, in our experiments, SHG is mainly influenced by two factors: symmetry breaking and plasmon-driven field enhancement. Corresponding to the Equation (1), the first effect changes the effective $\chi_{\text{eff}}^{\left(2\right)}$ , while the second one changes $E\left(\omega \right)$ (local field).

## 3 Conclusions

In conclusion, we demonstrate modulation of the SHG intensity in gold nanosphere dimers through symmetry breaking. The optical dark field characterization enables us to distinguish monomers and dimers, as well as symmetric and asymmetric dimers, and identify their axes at the single-particle level. Finally, while the near-field enhancement of the gold nanosphere dimers still dominates the amplification of SHG under on-resonant excitation, we can observe the symmetry-driven modulation of the SHG intensity under the off-resonant conditions. We anticipate an extension of our approach to other nanoplasmonic systems where nonlinear-optical properties can be tuned by tailoring the mesoscopic symmetry. Our findings emphasize the significance of symmetry breaking in nonlinear optics and have potential applications in the development of novel nanodevices for optical sensing, imaging, and communication technologies.

## Supplementary Material

Supplementary Material Details

## References

[1] You H. (2022). Accelerated pyro-catalytic hydrogen production enabled by plasmonic local heating of Au on pyroelectric BaTiO3 nanoparticles. *Nat. Commun.*.

[2] Chen J. (2022). Collective plasmon coupling in gold nanoparticle clusters for highly efficient photothermal therapy. *ACS Nano*.

[3] Shvalya V. (2022). Bacterial DNA recognition by SERS active plasma-coupled nanogold. *Nano Lett.*.

[4] Anker J. N., Hall W. P., Lyandres O., Shah N. C., Zhao J., Duyne R. P. V. (2008). Biosensing with plasmonic nanosensors. *Nat. Mater.*.

[5] Zijlstra P., Chon J. W., Gu M. (2009). Five-dimensional optical recording mediated by surface plasmons in gold nanorods. *Nature*.

[6] Amendola V., Pilot R., Frasconi M., Maragò O. M., Iatì M. A. (2017). Surface plasmon resonance in gold nanoparticles: a review. *J. Phys.: Condens. Matter*.

[7] Wang M. (2022). Plasmonic phenomena in molecular junctions: principles and applications. *Nat. Rev. Chem*.

[8] Zohar N., Chuntonov L., Haran G. (2014). The simplest plasmonic molecules: metal nanoparticle dimers and trimers. *J. Photochem. Photobiol. C*.

[9] Ahmadivand A., Semmlinger M., Dong L., Gerislioglu B., Nordlander P., Halas N. J. (2018). Toroidal dipole-enhanced third harmonic generation of deep ultraviolet light using plasmonic meta-atoms. *Nano Lett.*.

[10] Cha H., Yoon J. H., Yoon S. (2014). Probing quantum plasmon coupling using gold nanoparticle dimers with tunable interparticle distances down to the subnanometer range. *ACS Nano*.

[11] Tan S. F. (2018). *Molecular Electronic Control Over Tunneling Charge Transfer Plasmons Modes*.

[12] Camargo P. H. C., Rycenga M., Au L., Xia Y. (2009). Isolating and probing the hot spot formed between two silver nanocubes. *Angew. Chem.*.

[13] Lim D. K., Jeon K. S., Kim H. M., Nam J. M., Suh Y. D. (2010). Nanogap-engineerable Raman-active nanodumbbells for single-molecule detection. *Nat. Mater.*.

[14] Zhang Z., Deckert-Gaudig T., Singh P., Deckert V. (2015). Single molecule level plasmonic catalysis–a dilution study of p-nitrothiophenol on gold dimers. *Chem. Commun.*.

[15] Zhang C. (2016). Al–pd nanodisk heterodimers as antenna–reactor photocatalysts. *Nano Lett.*.

[16] Slablab A. (2012). Second-harmonic generation from coupled plasmon modes in a single dimer of gold nanospheres. *Opt. Express*.

[17] Franken P. A., Hill A. E., Peters C. W., Weinreich G. (1961). Generation of optical harmonics. *Phys. Rev. Lett.*.

[18] Butet J., Brevet P. F., Martin O. J. F. (2015). Optical second harmonic generation in plasmonic nanostructures: from fundamental principles to advanced applications. *ACS Nano*.

[19] Butet J. (2010). Optical second harmonic generation of single metallic nanoparticles embedded in a homogeneous medium. *Nano Lett.*.

[20] Butet J., Bachelier G., Russier-Antoine I., Jonin C., Benichou E., Brevet P. F. (2010). Interference between selected dipoles and octupoles in the optical second-harmonic generation from spherical gold nanoparticles. *Phys. Rev. Lett.*.

[21] Nappa J., Revillod G., Russier-Antoine I., Benichou E., Jonin C., Brevet P. F. (2005). Electric dipole origin of the second harmonic generation of small metallic particles. *Phys. Rev. B*.

[22] Nappa J., Russier-Antoine I., Benichou E., Jonin C., Brevet P. F. (2006). Second harmonic generation from small gold metallic particles: from the dipolar to the quadrupolar response. *J. Chem. Phys.*.

[23] Meier J., Zurak L., Locatelli A., Feichtner T., Kullock R., Hecht B. (2023). Controlling field asymmetry in nanoscale gaps for second harmonic generation. *Adv. Opt. Mater.*.

[24] Johnson J. C., Yan H., Schaller R. D., Petersen P. B., Yang P., Saykally R. J. (2002). Near-field imaging of nonlinear optical mixing in single zinc oxide nanowires. *Nano Lett.*.

[25] Nakayama Y. (2007). Tunable nanowire nonlinear optical probe. *Nature*.

[26] O’Brien K. (2015). Predicting nonlinear properties of metamaterials from the linear response. *Nat. Mater.*.

[27] Neely A. (2009). Ultrasensitive and highly selective detection of alzheimer’s disease biomarker using two-photon Rayleigh scattering properties of gold nanoparticle. *ACS Nano*.

[28] Li G.-C., Zhang Y. L., Jiang J., Luo Y., Lei D. Y. (2017). Metal-substrate-mediated plasmon hybridization in a nanoparticle dimer for photoluminescence line-width shrinking and intensity enhancement. *ACS Nano*.

[29] Canfield B. K., Kujala S., Jefimovs K., Turunen J., Kauranen M. (2004). Linear and nonlinear optical responses influenced by broken symmetry in an array of gold nanoparticles. *Opt. Express*.

[30] Husu H. (2008). Local-field effects in the nonlinear optical response of metamaterials. *Metamaterials*.

[31] Canfield B. K. (2007). Local field asymmetry drives second-harmonic generation in noncentrosymmetric nanodimers. *Nano Lett.*.

[32] Zhang Y., Wei C., Gao F., Lei D., Yang F. (2023). Revisiting the linear and nonlinear optical properties of nanoparticle-on-mirror–type plasmonic metasurfaces with transformation optics. *Phys. Rev. B*.

[33] Li G. C., Lei D., Qiu M., Jin W., Lan S., Zayats A. V. (2021). Light-induced symmetry breaking for enhancing second-harmonic generation from an ultrathin plasmonic nanocavity. *Nat. Commun.*.

[34] Kravets V. G., Kabashin A. V., Barnes W. L., Grigorenko A. N. (2018). Plasmonic surface lattice resonances: a review of properties and applications. *Chem. Rev.*.

[35] Yoon J. H., Selbach F., Langolf L., Schlücker S. (2018). Ideal dimers of gold nanospheres for precision plasmonics: synthesis and characterization at the single-particle level for identification of higher order modes. *Small*.

[36] Heuer-Jungemann A. (2019). The role of ligands in the chemical synthesis and applications of inorganic nanoparticles. *Chem. Rev.*.

[37] Jose J. (2022). Particle size-dependent onset of the tunneling regime in ideal dimers of gold nanospheres. *ACS Nano*.

[38] Yang L., Wang H., Fang Y., Li Z. (2016). Polarization state of light scattered from quantum plasmonic dimer antennas. *ACS Nano*.

[39] Yoon J. H., Selbach F., Schumacher L., Jose J., Schlücker S. (2019). Surface plasmon coupling in dimers of gold nanoparticles: experiment and theory for ideal (spherical) and nonideal (faceted) building blocks. *ACS Photonics*.

[40] Prodan E., Radloff C., Halas N. J., Nordlander P. (2003). A hybridization model for the plasmon response of complex nanostructures. *Science*.

[41] Nordlander P., Oubre C., Prodan E., Li K., Stockman M. (2004). Plasmon hybridization in nanoparticle dimers. *Nano Lett.*.

[42] Thomas K. G., Barazzouk S., Ipe B. I., Joseph S. T. S., Kamat P. V. (2004). Uniaxial plasmon coupling through longitudinal self-assembly of gold nanorods. *J. Phys. Chem. B*.

[43] Abou-Hamdan L., Li C., Haidar R., Krachmalnicoff V., Bouchon P., De Wilde Y. (2021). Hybrid modes in a single thermally excited asymmetric dimer antenna. *Opt. Lett.*.

[44] Jain P. K., Eustis S., El-Sayed M. A. (2006). Plasmon coupling in nanorod assemblies: optical absorption, discrete dipole approximation simulation, and exciton-coupling model. *J. Phys. Chem. B*.

[45] Ha J. W. (2015). Characteristic image patterns of single anisotropic plasmonic nanoparticles embedded in a gel matrix. *Nanoscale*.

[46] Zhang Q., Li G. C., Lo T. W., Lei D. Y. (2018). Polarization-resolved optical response of plasmonic particle-on-film nanocavities. *J. Opt.*.

[47] Johnson P. B., Christy R. W. (1972). Optical constants of the noble metals. *Phys. Rev. B*.

[48] Ulman A. (1996). Formation and structure of self-assembled monolayers. *Chem. Rev.*.

[49] Widrig C. A., Chung C., Porter M. D. (1991). The electrochemical desorption of n-alkanethiol monolayers from polycrystalline au and ag electrodes. *J. Electroanal. Chem.*.

[50] Slowinski K., Chamberlain R. V., Bilewicz R., Majda M. (1996). Evidence for inefficient chain-to-chain coupling in electron tunneling through liquid alkanethiol monolayer films on mercury. *J. Am. Chem. Soc.*.

[51] Miller C., Cuendet P., Graetzel M. (1991). Adsorbed.omega.-hydroxy thiol monolayers on gold electrodes: evidence for electron tunneling to redox species in solution. *J. Phys. Chem.*.

[52] Porter M. D., Bright T. B., Allara D. L., Chidsey C. E. D. (1987). Spontaneously organized molecular assemblies. 4. Structural characterization of n-alkyl thiol monolayers on gold by optical ellipsometry, infrared spectroscopy, and electrochemistry. *J. Am. Chem. Soc.*.

[53] Krasavin A. V., Ginzburg P., Zayats A. V. (2018). Free-electron optical nonlinearities in plasmonic nanostructures: a review of the hydrodynamic description. *Laser Photon. Rev.*.

[54] Berthelot J. (2012). Silencing and enhancement of second-harmonic generation in optical gap antennas. *Opt. Express*.

[55] Kleemann M. E. (2017). Revealing nanostructures through plasmon polarimetry. *ACS Nano*.

[56] Tserkezis C. (2015). Hybridization of plasmonic antenna and cavity modes: extreme optics of nanoparticle-on-mirror nanogaps. *Phys. Rev. A*.

