# Supplementary material for: Symmetry-breaking-induced off-resonance second-harmonic generation enhancement in asymmetric plasmonic nanoparticle dimers
Source: Nanophotonics. 2024 Jun 3;13(18):3337–46. doi: 10.1515/nanoph-2024-0118 (PMC11501254; doi:10.1515/nanoph-2024-0118)
Supplement: Supplementary file 1 — Supplementary Material Details [file j_nanoph-2024-0118_suppl_001.pdf]

Yaorong Wang, Zhiwei Peng, Yannick De Wilde, and Dangyuan Lei

Symmetry-Breaking-Induced Off-Resonance Second-Harmonic Generation Enhancement in Asymmetric Plasmonic Nanoparticle Dimers (Supporting Information)

1. Sample fabrication

Highly spherical gold nanoparticles (NanoSeedz) of varying diameters (~60 nm, ~80 nm, and ~100 nm) were sequentially assembled on a substrate to prepare dimers. The gold nanosphere were coated with a surfactant layer of cetyltrimethylammonium bromide (CTAB). As shown in Figure S1(a), the synthesis of the dimers comprises five steps.

Step 1: The starting materials for the dimers are CTAB-capped gold nanoparticles and cleaned glass. A glass slide (25 mm × 12 mm) cleaned with a hot RBS detergent solution (15%, 90 °C) was immersed in a CTAB solution (5×10^{-6} m, 5 mL) containing gold nanoparticles (5×10^{-12} m) for 17 h at 30 °C. All subsequent steps were performed at 30 °C. The glass substrate was given a negative charge by washing them in RBS detergent solution. The surface charge of nanoparticles is mainly regulated by surface ligands: the common positive charge ligands like CTAB and negatively charged ligands like polyvinylpyrrolidone (PVP). But in different solvents, the surface electrical properties of nanoparticles with the same surface ligands can be significantly modified. For example, CTAB-capped gold exhibits positive electrical properties in aqueous solution, but significantly negative electrical properties in acetonitrile solution. In this step, the first nanoparticle is made to attach to the glass substrate via the electrostatic interaction between the substrate and the positively charged CTAB bilayer surrounding the nanoparticle. We refer to the gold nanoparticles as the first and second nanoparticle according to the order in which they were added to the system.

Step 2: The glass slide was washed with water and ethanol (ACS grade, Anaqua). For a well-ordered linker self-assembled monolayer (SAM) on the surface of the nanoparticles, the washed glass was exposed to a mixture of an ethanolic solution of the 1,8 octanedithiol (C8, ≥ 98%, Aladdin) and an aqueous solution of sodium bromide (NaBr, ≥ 99%, Aladdin)(v/v 250:1). The final concentration of both alkanedithiol and NaBr was 1×10^{-3} m. The addition of inorganic salts to the aqueous suspension medium such as NaBr or the use of organic suspension media such as

acetonitrile increases the critical micelle concentration of CTAB on the metal surface.[1,2] Importantly, only the combination of both inorganic salt and organic suspension medium efficiently destroys the CTAB bilayer, so that a well-ordered linker SAM forms within 1 h.

Step 3: The glass substrate coated with the nanoparticles was washed using ethanol. For the synthesis of the dimers, the glass slide was dipped into 5 mL of acetonitrile (HPLC grade, RciLabscan) containing nanoparticles (20×10^{-12} m) and NaBr (200×10^{-6} m) for 5 h. If an asymmetric dimer has to be prepared, gold spheres with larger diameters need to be added at this step.

Step 4: For stabilizing the surface of the secondly added nanoparticles, the glass substrate was washed using ethanol and immersed for 1 h in a mixture of an ethanolic solution of (11-mercaptoundecyl)-N,N,N-trimethylammonium bromide (MUTAB, ≥ 90%, Aldrich) and an aqueous solution of NaBr (v/v 250:1). The final concentration of MUTAB is 1×10^{-3} m.

Step 5: Dimers were removed from the glass substrate using sonication for 30 s. For the stabilization of the newly exposed area of ideal dimers upon the sonication, an ethanolic solution of MUTAB (10×10^{-6} m) was employed as a dispersion medium.

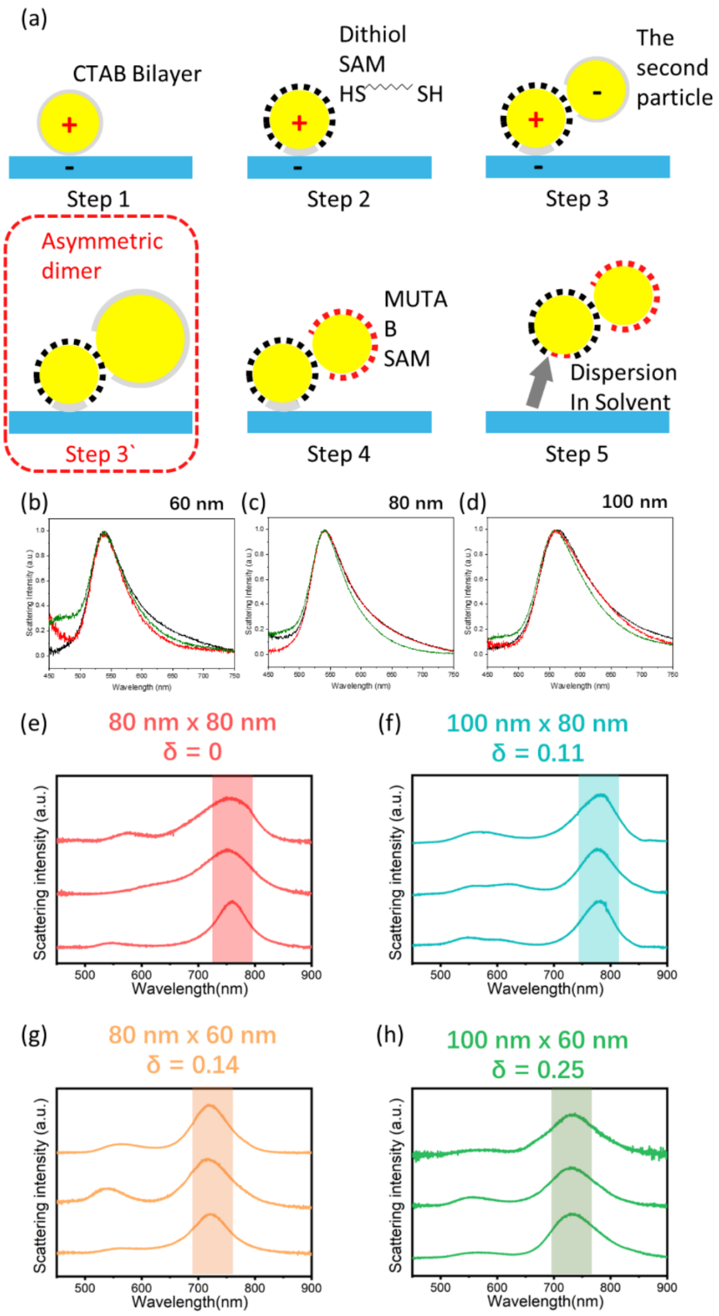

Figure S1. (a) Schematic diagram of the assembled dimer. Single-particle dark-field scattering spectra of (b) 60 nm monomers, (c) 80 nm monomers, (d)100 nm monomers, (e) 80 x 80 nm dimers, (f) 100 x 80 nm dimers, (g) 80 x 60 nm dimers, (h),100 x 60 nm dimers. The LBDP band is highlighted.

2.Numerical methods for SHG simulations

In the nonlinear simulations, we adopted the perturbative approach within the undepleted pump approximation, which is justified by the fact that the SHG field intensity generated by a plasmonic nanostructure is always orders of magnitude weaker than that of the excitation field. Under this approximation, the SHG field does not couple back to the pump field and, therefore, the nonlinear process can be decomposed into two linear scattering processes: firstly, the fundamental fields were solved with the finite element solver in the frequency domain and the calculated fields were used to find the SHG polarization source currents that are responsible for the far-field SHG emission. Although both the free and bound electrons in metals can give rise to the second-order nonlinear process, it has been proven that only the former is responsible for the dominant SHG response in the visible and near- infrared spectral range.[3] The SHG polarization currents related to free electrons can be further decomposed into the volume and surface contributions. Here, we only consider the second-harmonic polarization which is normal to the metal surface for it plays the dominant role in the SHG.[4] In this case, the SHG source currents based on the free-electron hydrodynamic model can be found as[5]

$$\hat{\mathbf{n}} \cdot \mathbf{J}_{\mathrm{surf}} = i\frac{n_0 e^3}{2m_*^2}\frac{3+\varepsilon_{\mathrm{EF}}(\omega)}{(\omega+i\gamma)^2(2\omega+i\gamma)}E_\perp^2(\omega) \tag{1}$$

where $\hat{\mathbf{n}}$ is the unit vector normal to the metal surface, $\mathbf{J}_{\mathrm{surf}}$ is the surface current, n_0 is the free-electron density in gold, m_* represents the effective electron mass, e is the elementary charge, γ is the electron gas collision frequency in gold, $\varepsilon_{\mathrm{EF}}$ is the bulk gold permittivity at the fundamental wavelength, and $E_\perp(\omega)$ is the normal component of the local fundamental field at the metal surface. Due to the limited penetration of the electromagnetic fields into the bulk gold, the induced nonlinear polarization occurs mainly in the sub-nanometer-thick surface layer. Considering the rotational symmetry of a nanoparticle dimer, the simulation configuration can be simplified into a two-dimensional frame for which the meshing for the surface layer, where the SHG source is located, can be scaled down to the level of 0.1 Å (Figure S2 (a)).

By integrating $P^0_{z,2\omega}$ show in Figure 3 in main text, we can get a rough P(2ω). We did not calculate the results in all spaces but only selected this small area because outside the area, $P^0_{z,2\omega}$ will decay rapidly, so it can be ignored. By changing the wavelength of the fundamental frequency, we can get the relationship between $|P(2\omega)|^2$ and wavelength.

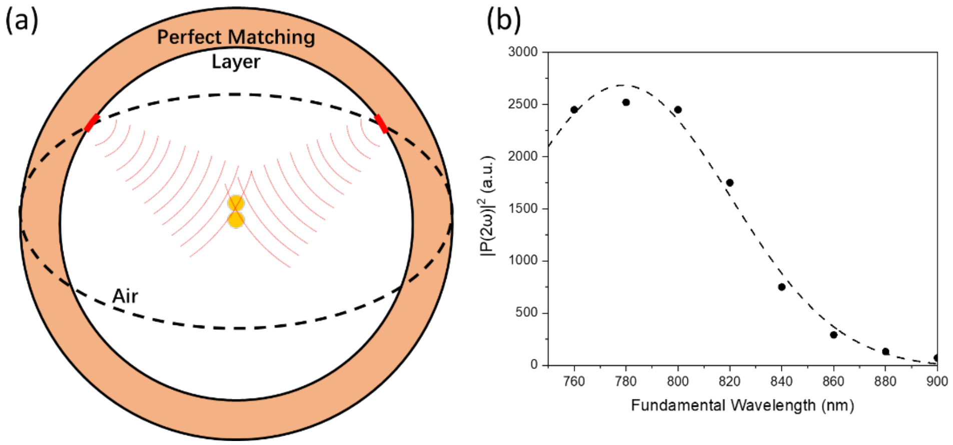

Figure S1 (a) Simulation domains for a gold nanoparticle dimer. The excitation source is modeled by the current oscillator with the oscillation direction tangential to the inner surface of the perfectly matching layer. Such excitation configuration can preferentially excite the gap plasmon modes of the gold nanoparticle dimer. (b) Spectra of the P(2ω) simulated for the dimer with δ = 0.11 at different excitation wavelengths.

3. Linear optical properties of dimers

Optical microscopy and spectroscopy were performed with an Olympus microscope equipped with a standard dark-field optical module using a white light source (Olympus, 12V/100 W Quartz Halogen) and a 100× dark-field objective (LMPlanFLN, Olympus, NA=0.8). Dark-field images and the spectra of individual nanostructures were recorded respectively using a color CCD camera (OPLENIC) and a spectrograph (Andor SR500i). The individual nanosphere monomers and dimers on a silica substrate show significantly different scattering responses. These distinct properties can thus be employed to identify the nanoparticle dimers from the monomers and to determine their in-plane orientations with the polarization-resolved dark-field microscopy.

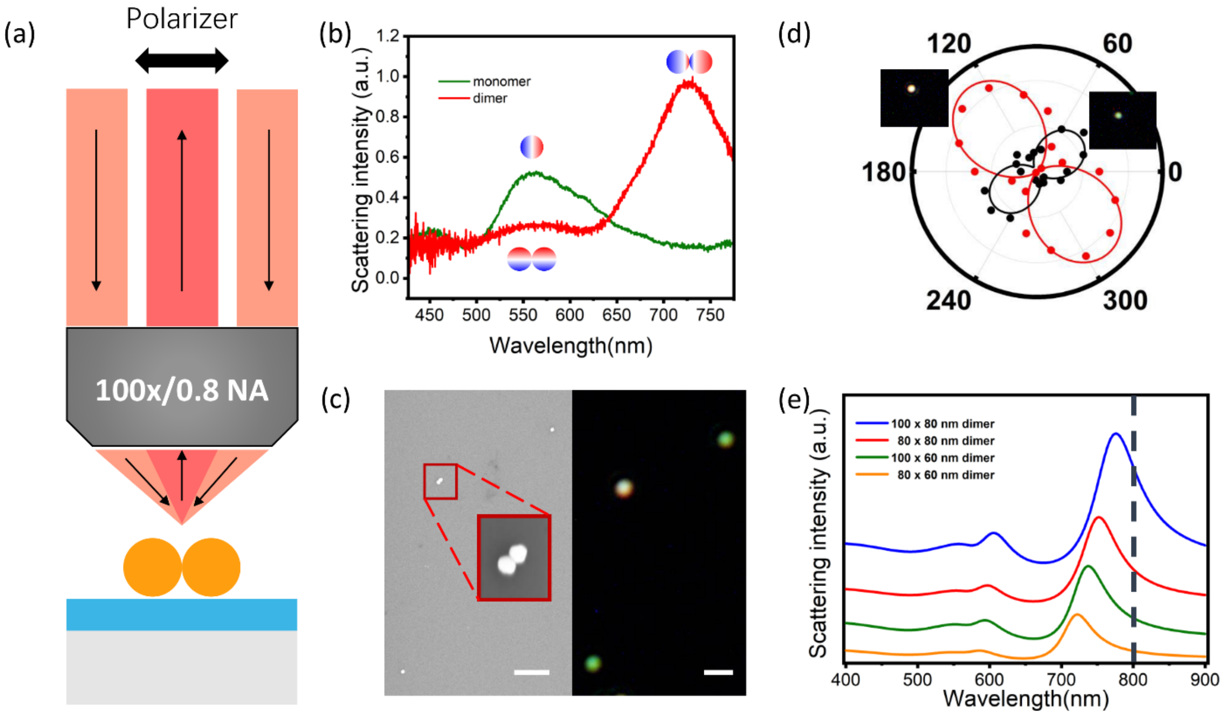

Figure S2 (a) Experimental schematic for DF. (b) Scattering spectrum of a gold nanoparticle monomer and dimer on the substrate. Insets show the diagram of their modes. (c) SEM micrograph (left) of two nanosphere monomers and a nanosphere dimer (enclosed by the red square). The right panel shows the corresponding optical dark-field image for the same sample area. The scale bars in both images are 1 μm. (d) The dimer exhibits polarization-dependent scattering intensity patterns, which can be employed to determine its orientation. The inset shows DF images with different polarization orientations. (e) Simulated scattering spectra of different dimers.

4. Evaluation of laser damage to the gold nanoparticle dimers

Since the gap plasmon resonance of dimer is sensitive to the gap morphology and distance, we rely on the comparison of the measured scattering spectra of single nanostructures before and after the laser irradiation. For the laser irradiation power ~0.6 mW, most of the nanoparticle dimers show scattering spectra as in Figure S4. The peak shift of less than 10 nm indicates negligible gap variation. In our experiments, the peak intensity of a single pulse is about 5 GW/cm^2, and the duration of each measurement is 90 s.

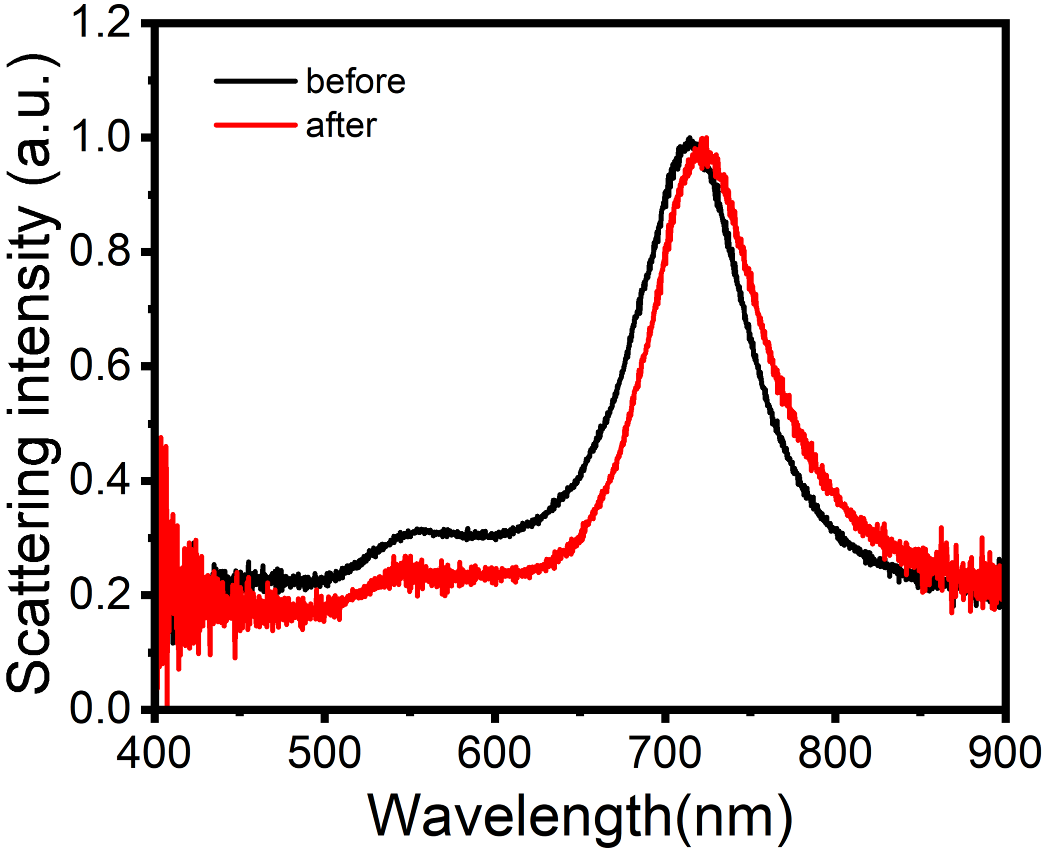

Figure S4 Representative scattering spectra of a nanoparticle dimer measured before and after the SHG measurements.

5. Evaluation of the second harmonic conversion efficiency

To quantitatively evaluate the SHG efficiency of the nano-dimer system, we define the conversion efficiency as $\eta_{SHG} = P_{2\omega}/P_{\omega}^2$, The Pω is recorded using a power meter in front of the sample stage. The P2ω can be experimentally evaluated by taking into account several parameters, i.e., the delivery efficiency of the objective lens, transmission coefficients of all optical elements, and the quantum efficiency of the detector, respectively. Parameters acquired with this analysis are added in Supporting Information. Taking dimer with δ= 0.25 under 900 nm excitation as an example, estimated conversion efficiencies of nominally 5.3×10^{-9}.

Table 1 Analysis of the SHG efficiency

Objective collection efficiency	Delivery efficiency	Detector Collection Efficiency
0.91	0.2	0.271
$P_{2\omega}$ (W)	P_{ω} (W)	$\eta_{SHG}=P_{2\omega}/P_{\omega}^{2}$
1.5×10^{-5}	53	5.3×10^{-9}

6. Polarization properties of the SHG signals

The polar plot of SHG of dimer with δ= 0.25 shows a double-lobed pattern similar to the bonding dipole pattern. This indicates that the polarization of the nonlinear emission of the dimer is dominated by the dipole modes, exhibiting maximum and minimum intensity at 0° (180°) and 90° (270°), respectively.

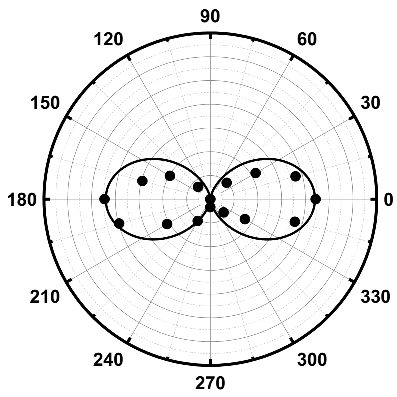

Figure S5 Polarization properties of the SHG signals.

References

[1] K. Liu, Y. Zheng, X. Lu, T. Thai, N. A. Lee, U. Bach, J. J. Gooding, "Biocompatible gold nanorods: One-step surface functionalization, highly colloidal stability, and low cytotoxicity," *Langmuir,* vol. 31, no. 1, pp. 4973–4980, 2015.

[2] D. Nepal, K. Park, R. A. Vaia, "High-Yield Assembly of Soluble and Stable Gold Nanorod Pairs for High-Temperature Plasmonics," *Small*, vol. 8, no. 1, pp.1013–1020, 2012.

[3] M. Scalora, M. A. Vincenti, D. de Ceglia, V. Roppo, M. Centini, N. Akozbek, M. J. Bloemer, "Second-and third-harmonic generation in metal-based structures," *Phys. Rev. A,* vol. 82, no.1, p. 043828, 2010.

[4] G. Marino, P. Segovia, A. V. Krasavin, P. Ginzburg, N. Olivier, G. A. Wurtz, A. V. Zayats, "Second-harmonic generation from hyperbolic plasmonic nanorod metamaterial slab," *Laser Photonics Rev.,* vol. 12, no. 1, p. 1700189, 2018.
